# Altered vitamin D_3_ metabolism in the ovary and periovarian adipose tissue of rats with letrozole-induced PCOS

**DOI:** 10.1007/s00418-020-01928-z

**Published:** 2020-10-23

**Authors:** Malgorzata Grzesiak, Gabriela Burzawa, Patrycja Kurowska, Klaudia Blaszczyk, Agata Szlaga, Anna Blasiak, Andrzej Sechman, Agnieszka Rak

**Affiliations:** 1 Department of Endocrinology, Institute of Zoology and Biomedical Research, Jagiellonian University in Krakow, Gronostajowa 9, 30-387 Krakow, Poland; 2 Department of Animal Physiology and Endocrinology, University of Agriculture in Krakow, Krakow, Poland; 3 Department of Physiology and Toxicology of Reproduction, Institute of Zoology and Biomedical Research, Jagiellonian University in Krakow, Krakow, Poland; 4 Department of Neurophysiology and Chronobiology, Institute of Zoology and Biomedical Research, Jagiellonian University in Krakow, Krakow, Poland

**Keywords:** CYP27B1, CYP24A1, Vitamin D_3_ receptor, Polycystic ovary syndrome, Periovarian adipose tissue

## Abstract

Vitamin D_3_ (VD_3_) plays an important role in the ovary and its deficiency is associated with ovarian pathologies, including polycystic ovary syndrome (PCOS). However, there is no data related to VD_3_ metabolism in the ovary during PCOS. Herein, we investigated differences in the expression of VD_3_ receptor (VDR) and key VD_3_ metabolic enzymes, 1α-hydroxylase (CYP27B1) and 24-hydroxylase (CYP24A1), in the ovary and periovarian adipose tissue (POAT) of control (proestrus and diestrus) and PCOS induced by letrozole rats. *Vdr*, *Cyp27b1* and *Cyp24a1* mRNA expression was determined, their protein abundance was examined and immunolocalized. Furthermore, VD_3_ metabolite concentrations in plasma (25OHD) and tissues (ovary and POAT; 1,25(OH)_2_D_3_), and plasma calcium level were determined. 25OHD concentration decreased markedly in letrozole-treated rats in comparison with controls, whereas calcium concentration did not vary among the examined groups. The amount of 1,25(OH)_2_D_3_ decreased in both ovary and POAT of PCOS rats. In the ovary, we found decreased *Cyp27b1* and increased *Vdr* mRNA expression in letrozole-treated and diestrus control group. Corresponding protein abundances were down-regulated and up-regulated, respectively but only following letrozole treatment. In POAT, only *Cyp27b1* transcript level and CYP27B1 protein abundance were decreased in letrozole-treated rats. VDR was immunolocalized in healthy and cystic follicles, while CYP27B1 and CYP24A1 were found exclusively in healthy ones. Concluding, our results provide the first evidence of disrupted VD_3_ metabolism in the ovary and POAT of PCOS rats. The reduced 1,25(OH)_2_D_3_ concentration in those tissues suggests their contribution to VD_3_ deficiency observed in PCOS and might implicate in PCOS pathogenesis.

**Electronic supplementary material:**

The online version of this article (10.1007/s00418-020-01928-z) contains supplementary material, which is available to authorized users.

## Introduction

It is widely accepted that vitamin D_3_ (VD_3_) regulates calcium and phosphorus homeostasis to ensure bone health (DeLuca 2004). However, due to the broad distribution of VD_3_ receptor (VDR) in the organism, VD_3_ effects extend to various tissues, including female reproductive organs. Recent studies also reveal that VD_3_ insufficiency/deficiency associates with many reproductive disorders, such as endometriosis, uterine fibroids, premature ovarian failure, ovarian cancer and polycystic ovarian syndrome (PCOS) (Dabrowski et al. 2015; Muscogiuri et al. 2017).

In humans and animals, the main source of circulating active VD_3_ is its endogenous synthesis in the skin upon ultraviolet-B irradiation. In keratinocytes, 7-dehydrocholesterol is converted to previtamin D_3_, which is metabolized in the liver by 25-hydroxylases (*e.g.* CYP2R1, CYP27A1) to 25OHD_3_, and further in the kidney by 1α-hydroxylase (CYP27B1) to the bioactive hormone 1,25(OH)_2_D_3_. Both 1,25(OH)_2_D_3_ and 25OHD_3_ are inactivated by a mitochondrial 24-hydroxylase (CYP24A1) (Bikle 2014). To date, the ovary has been identified as an extrarenal site of CYP27B1 expression and VDR is abundant in ovarian cells, suggesting local VD_3_ metabolism and action within the mammalian ovary (Herian et al. 2018; Xu et al. 2018). Indeed, there are numerous reports of positive VD_3_ effects on folliculogenesis and steroidogenesis. In details, VD_3_ was shown to increase the synthesis of anti-Müllerian hormone (AMH), maintaining the ovarian reserve (Irani and Merhi 2014). In addition, Xu et al. (2018) reported a survival and development of preantral and antral follicles in rhesus macaque following VD_3_ exposure in vitro. Moreover, VD_3_ was found to stimulate progesterone and 17β-estradiol secretion by human granulosa cells (Parikh et al. 2010; Merhi et al. 2014). On the other hand, hypovitaminosis D_3_ has been linked to several ovarian pathologies such as PCOS (He et al. 2015).

PCOS is a common disorder affecting women of reproductive age (Fauser et al. 2012). In general, two criteria, such as oligoovulation/anovulation, hyperandrogenism and polycystic ovaries on ultrasound, must be met for PCOS diagnosis (Azziz et al. 2006). However, its phenotype is accompanied by a much wider range of reproductive (ovulatory dysfunction, polycystic ovarian morphology, infertility), endocrine (hyperandrogenism, disturbed gonadotropins level) and metabolic (glucose metabolism, insulin sensitivity, lipid profile) features (Fauser et al. 2012; Krul-Poel et al. 2018). Recent clinical and experimental studies have reported that VD_3_ deficiency among women contributes to various disturbances associated with PCOS (Muscogiuri et al. 2017). To our knowledge, low serum VD_3_ status causes dysregulation in calcium metabolism that may inhibit follicle maturation and ovulation in women with PCOS (Thys-Jacobs et al. 1999). The decreased VD_3_ level also reduces the activity and expression of aromatase, which impairs conversion of androgens to estrogens. In turn, increased androgen concentration blocks follicular maturation before ovulation and leads to ovarian cyst appearance (Lorenzen et al. 2017). Furthermore, VD_3_ deficiency is significantly associated with a higher insulin resistance and a lower level of high-density lipoproteins in PCOS patients (Krul-Poel et al. 2018). By this time, VD_3_ supplementation has been shown to improve menstrual regularity and ovulatory dysfunction as well as decrease serum androgens level and increase insulin sensitivity in women with PCOS (Karadağ et al. 2018; Shojaeian et al. 2019). However, information about local ovarian VD_3_ metabolism in PCOS is still lacking. In the present study, we hypothesize that the VD_3_ metabolic system differs between normal and polycystic ovaries.

This research aimed to determine whether VD_3_ metabolic homeostasis is altered in the ovary and surrounding periovarian adipose tissue (POAT) of rats with PCOS induced by aromatase inhibitor letrozole (Kafali et al. 2004). To meet this goal we examined: (1) plasma 25OHD and calcium concentrations in control and PCOS rats, (2) 1,25(OH)_2_D_3_ concentration in ovarian and POAT homogenates, (3) *Vdr*, *Cyp7b1* and *Cyp24a1* mRNA expression, and (4) VDR, CYP27B1 and CYP24A1 proteins abundance, and (5) immunolocalization in the ovary and POAT obtained from control and PCOS rats.

## Materials and methods

### Animals

Six-week-old female Wistar rats (mean body weight (BW) at the beginning of the experiment 191.375 ± 7.87 g) were purchased from Faculty of Pharmacy Jagiellonian University Medical College (Krakow, Poland) and Mossakowski Medical Research Centre Polish Academy of Sciences (Warsaw, Poland). Animals were housed in controlled conditions of temperature, humidity and light (12 h light: 12 h dark cycle) with ad libitum availability of food and water. Rats were allowed to acclimatize for one week before treatment. All experimental protocols listed herein were approved by the Local Animal Ethical Committee, Poland (permit no. 277/2019).

PCOS was induced in rats by administration of nonsteroidal aromatase inhibitor letrozole (Sigma-Aldrich, St. Louis, MO, USA) (Kafali et al. 2004; Kalamon et al. 2020). For PCOS induction, 48 rats were randomly assigned into the control (*n* = 32) and letrozole-treated (*n* = 16) groups. A daily treatment regime of 21 days included oral administration through gavage of either 2% (v/v) dimethyl sulfoxide (DMSO; Sigma-Aldrich) in rapeseed oil (1 ml/kg BW) in the first group or letrozole (1 mg/kg BW) dissolved in 2% DMSO in rapeseed oil in the second group. Rats were weighed daily for 21 consecutive days of the experiment. After 21 days of treatment, BW, estrous cyclicity, plasma testosterone (T) and 17β-estradiol (E_2_) levels as well as ovarian histology were analyzed to verify the development of PCOS.

### Assessment of estrous cyclicity

Estrous cyclicity was monitored by microscope analysis of vaginal epithelial cell smears. Smears were stained using May-Grundwald and Giemsa procedure (Kolchem, Lodz, Poland). Proestrus was characterized by the presence of predominantly nucleated and some cornified epithelial cells, estrus as mostly cornified cells, metestrus as some cornified epithelial cells and primarily leukocytes and diestrus as primarily leukocytes (Marcondes et al. 2002). As a control groups, animals from proestrus and diestrus phases were chosen (*n* = 16 per each group) (Baravalle et al. 2006).

### Blood and tissues samples

Collection of blood and tissue samples was performed between 1:00 and 3:00 pm during proestrus and diestrus stages of the estrous cycle of control rats and from acyclic PCOS-induced rats. Animals were deeply anesthetized by inhalation of 4% isoflurane in an enclosed vessel. Blood was withdrawn through the orbital sinus in a tube and centrifuged at 500* g* for 15 min at room temperature. Plasma was separated and immediately stored at  – 20 °C for further analyses. Ovaries and POAT were excised from all animals. Separate ovary and surrounding POAT was snap-frozen in liquid nitrogen for RNA and protein isolation, 1,25(OH)_2_D_3_ concentration measurement, as well as was fixed in formalin (10% buffered formalin for ovaries and zinc-formalin for POAT) for routine histology (hematoxylin–eosin staining) and immunohistochemistry.

### Antibody validation

Antibody specificity (anti-VDR, anti-CYP27B1 and anti-CYP24A1) was determined by Western blot analysis performed on lysates from rat kidney (positive control tissue for VDR, CYP27B1 and CYP24A1) and rat heart (negative control tissue for CYP27B1 and CYP24A1) in comparison to lysates from rat ovary. Analyzed proteins were shown in kidney and ovarian samples as specific bands at approximately 48 kDa (VDR), 56 kDa (CYP27B1) and 59 kDa (CYP24A1) (Supplementary File 1). Furthermore, the analysis of non-specific binding of secondary antibodies by omission of primary antibodies was conducted (Supplementary File 2). Validation of primary antibodies was also performed by immunohistochemistry on rat kidney sections as a positive control. The incubation of sections with respective non-immune serum (rabbit: NI01 or goat: NI02; Calbiochem, Darmstadt, Germany) was used as a negative control (Supplementary File 3).

According to policy of antibody validation we applied independent antibody strategy with other antibodies against VDR (MA1-710; Thermo Scientific, Rockford, IL, USA) and CYP27B1 (PA5-79,128; Thermo Fisher Scientific) recognizing different region of target proteins when compared to primary antibodies used in the present study. The expression pattern generated by two independent antibodies toward VDR and CYP27B1 proteins yield correlated signals across loading samples, suggesting that both antibodies recognize the intended target (Supplementary File 4).

The anti-CYP24A1 antibody used in the present study was specific for rat and validated on rat tissues following manufacturer’s recommendation. It recognizes 153–514 amino acids sequence of human CYP24A1, which is homologous with rat in 86%. Furthermore, the specificity of this antibody was confirmed by Western blot and immunohistochemistry as shown in Supplementary Files 1 and 3.

### Ovarian and POAT histology

Paraplast-embedded ovaries and POAT from controls and letrozole-treated group were cut into 5 µm-thick sections and mounted on 3′3′-aminopropyl-triethoxysaline-coated (Sigma-Aldrich) slides. After deparaffinization and rehydration, tissue sections were stained with hematoxylin QS (Vector Laboratories, Burlingame CA, USA) and eosin Y (Sigma-Aldrich). Next, stained slides were dehydrated, mounted in DPX (Sigma-Aldrich) and coverslipped. Digital images were collected using an Axio Scope A1 microscope with EC Epiplan-NEOFLUAR objectives (Carl Zeiss, Jena, Germany) equipped with an Axiocam 503 color camera (2.83 megapixel: 1936 (H) × 1460 (V); pixel size 4.54 μm × 4.54 μm) with ZEN 2.3 pro software (Carl Zeiss, Jena, Germany).

Periovarian adipocyte size was measured according to Benrick et al. (2017). The quantification was conducted using ImageJ software (National Institutes of Health, Bethesda, MD, USA) and five representative micrographs per animal were analyzed. Following transformation to a 16-bit grayscale and setting the threshold to exclude anomalies such as blood vessels, the micrographs were transformed to black-and-white binary images and broken adipocyte plasma membranes were mended by applying the watershed function. Adipocytes were defined by circularity and cell area was measured in relation to a scale bar.

### T and E_2_ plasma concentration

T and E_2_ concentrations were determined in peripheral blood plasma using commercially available enzyme-linked immunosorbent assay kits (DRG MedTek, Warsaw, Poland). The sensitivity of each assay was 0.083 ng/mL for T and 9.714 pg/mL for E_2_, with ranges of 0–16 ng/mL, and 0–2000 pg/mL, respectively. The intra- and inter-assay coefficients of variation for T were 3.28% and 6.71%, respectively, and those for E_2_ were 2.71% and 6.72% respectively. All analyses were performed in duplicate.

### Calcium plasma concentration

Plasma calcium concentration was measured using commercial colorimetric assays (Pointe Scientific, Brussels, Belgium) according to the manufacturer’s protocol. Detection limit was 1 mg/dL. The intra- and inter-assay coefficients of variation were 1.1% and 2.0%, respectively. All analyses were performed in duplicate.

### 25OHD plasma concentration

The concentration of total 25OHD (25OHD_3_ and 25OHD_2_) in peripheral blood plasma was determined using a radioimmunological commercial kit (25OH-Vitamin D total-RIA-CT; DIAsourceImmunoAssays, Louvain-la-Neuve, Belgium), following the manufacturer's instruction. The concentration of 25OHD was measured using ^125^I-labeled hormone, standards and test tubes coated with an appropriate monoclonal antibody. Assay limit of detection was 5.67 pg/mL and the intra- and inter-assay coefficients of variation were 5.9% and 7.4%, respectively. All analyses were performed in duplicate.

### 1,25(OH)_2_D_3_ concentration in ovarian tissue and POAT

The concentration of 1,25(OH)_2_D_3_ in homogenates of ovaries and POAT was determined using a radioimmunological commercial kit (1,25(OH)_2_-Vitamin D-RIA-CT; DIAsourceImmunoAssays, Louvain-la-Neuve, Belgium). Prior to radioimmunoassay, fragments of ovaries and POAT from controls as well as letrozole-treated rats were weighed, homogenized in liquid nitrogen and dissolved in 0.1 M phosphate buffered saline (w/v) (PBS; pH 7.4). Following the manufacturer's protocol, the concentration of 1,25(OH)_2_D_3_ was measured using ^125^I-labeled hormone, standards and test tubes coated with an appropriate antibody. Assay limit of detection was 2.88 pg/mL and the intra- and inter-assay coefficients of variation were 7.4% and 11.3%, respectively. 1,25(OH)_2_D_3_ concentration in ovaries and POAT was expressed as ng per mg of examined tissue. All analyses were performed in duplicate.

### Quantitative real-time PCR analysis

Total cellular RNA was extracted from frozen ovarian and POAT samples using TRI Reagent solution (Ambion, Austin, TX, USA) according to the manufacturer's protocol. The quality and quantity of RNA were evaluated by measuring A260/A280 ratio using a NanoDrop™Lite Spectrophotometer (Thermo Scientific, Wilmington, DE, USA). Total RNA (1 µg) was used as a template in cDNA synthesis with High-Capacity cDNA Reverse Transcription Kit (Applied Biosystems, Foster City, CA, USA). The resulting cDNA was used for quantitative PCR using TaqMan Gene Expression Master Mix (Applied Biosystems) and rat-specific TaqMan Gene Expression Assays (Applied Biosystems) for *Vdr* (Rn00690616_m1), *Cyp27b1* (Rn00587137_m1) and *Cyp24a1* (Rn01423143_m1) following manufacturers' instructions. Real-time PCR reactions were conducted in duplicate with StepOne™ Real-Time PCR System (Applied Biosystems) according to the recommended cycling program (2 min at 50 °C, 10 min at 95 °C, 40 cycles of 15 s at 95 °C, and 1 min at 60 °C). Amplification of contaminating genomic DNA was checked by control experiments in which reverse transcriptase was omitted during the reverse transcription step. Relative mRNA quantification data were analyzed using the real-time PCR Miner algorithm (Zhao and Fernald 2005). The real-time PCR data obtained for *Vdr*, *Cyp27b1* and *Cyp24a1* were normalized to endogenous control, glyceraldehyde-3-phosphate dehydrogenase (*Gapdh*; assay ID: Rn01775763_g1).

### Western blot analysis

Extraction and quantification of proteins as well as Western blot analysis were performed as described previously (Rak-Mardyła et al. 2013). Proteins (50 μg/mL) were reconstituted directly in appropriate amounts of sample buffer, separated by12% SDS-PAGE using Mini-Protean TGX System Precast Protein Gels (Bio-Rad, Hercules, CA, USA) and then transferred to Trans-Blot Turbo Mini PVDF Transfer membranes (Bio-Rad). The membranes were washed and blocked in 0.02 M Tris-buffered saline containing 5% (w/v) bovine serum albumin (BSA) and 0.1% (v/v) Tween 20 and incubated overnight at 4 °C with primary antibodies: rabbit polyclonal anti-VDR (dilution 1:1000; cat. no 12550, Cell Signaling Technology, Danvers, MA, USA), goat polyclonal anti-CYP27B1 (dilution 1:200; cat. no sc-49643, Santa Cruz Biotechnology, Dallas, TX, USA), rabbit polyclonal anti-CYP24A1 (dilution 1: 1000: cat. no PA5-79,127, Thermo Fisher Scientific). Next, the membranes were washed with TBS (Tris-buffered saline) containing 0.1% (v/v) Tween 20 and incubated for 1 h with secondary horseradish peroxidase-conjugated goat anti-rabbit (dilution 1:1000; Cell Signaling Technology) or donkey anti-goat (dilution 1:1000; Santa Cruz Biotechnology) antibodies. Each membrane was stripped and reprobed with monoclonal mouse anti-β-actin antibody (1: 5000; Sigma-Aldrich) followed by horseradish peroxidase-conjugated horse anti-mouse antibody (dilution 1:1000; Cell Signaling Technology). Signals were detected by chemiluminescence using WesternBright Quantum (Advansta Inc., Menlo Park, CA, USA) and visualized using the Chemidoc XRS + System (Bio-Rad). All visible bands were densitometrically quantified and normalized to their corresponding β -actin bands using ImageJ software with the “Gel Analysis” functions. Semi-quantitative analysis was performed for three separately repeated experiments.

### Immunohistochemistry

Immunohistochemistry was performed as previously described (Szołtys et al. 2010; Grzesiak et al. 2019). Briefly, sections were deparaffinized, rehydrated and subjected to microwave (750 W) antigen retrieval in 0.01 M (w/v) citrate buffer (pH 6.0) for 12 min. Endogenous peroxidase activity was blocked with 0.3% (v/v) H_2_O_2_ in TBS for 20 min. To block non-specific binding sites, sections were incubated in 5% (v/v) normal rabbit or goat serum in TBS for 40 min. Sections were then incubated overnight with primary antibodies (for specification see "Western blot analysis") as follows: anti-VDR (dilution 1:50), anti-CYP27B1 (dilution 1:50) and anti-CYP24A1 (dilution 1:300). Next, slides were incubated with secondary biotinylated goat anti-rabbit (dilution 1:300; Vector Laboratory, Burlingame, CA, USA) or horse anti-goat (1:300; Vector Laboratory) antibodies for 1.5 h at room temperature, and avidin–biotin-peroxidase complex (Vectastain Elite ABC Reagent, Vector Laboratories) for 40 min at room temperature. Bound antibody was visualized with TBS containing 0.01% (v/v) H_2_O_2_, 0.05% (w/v) 3,3′-diaminobenzidine (DAB; Sigma-Aldrich) and 0.07% (w/v) imidazole for 1 min. Negative controls were performed by substituting the primary antibody with non-immune rabbit (NI01; Calbiochem) or goat (NI02; Calbiochem) IgGs. Sections were dehydrated, cleared in xylene and mounted in DPX (Sigma-Aldrich). Selected slides were photographed using an Axio Scope A1 microscope and an Axiocam503 color camera with ZEN 2.3 pro software.

### Statistical analysis

Statistical analyses were performed using Statistica v.13.1 software (StatSoft, Inc, Tulsa, OK, USA). All data are presented as the overall mean ± standard error of the mean (SEM), and differences were considered statistically significant at the 95% confidence level.

(*P* < 0.05). To verify the normal distribution of data the Shapiro–Wilk and the Lilliefors tests were applied. Data on T and E_2_, 25OHD, 1,25(OH)_2_D_3_ and calcium concentrations were analyzed by one-way ANOVA followed by Tukey post hoc test; BW results were analyzed by Student’s *t* test. Data from real-time PCR and Western blot analyses showed a lack of normal distribution and so the nonparametric Kruskal–Wallis test was applied and the differences between individual groups determined by post hoc Dunn's multiple comparison test.

## Results

### Effect of letrozole administration on rat BW

**Fig. 1:**
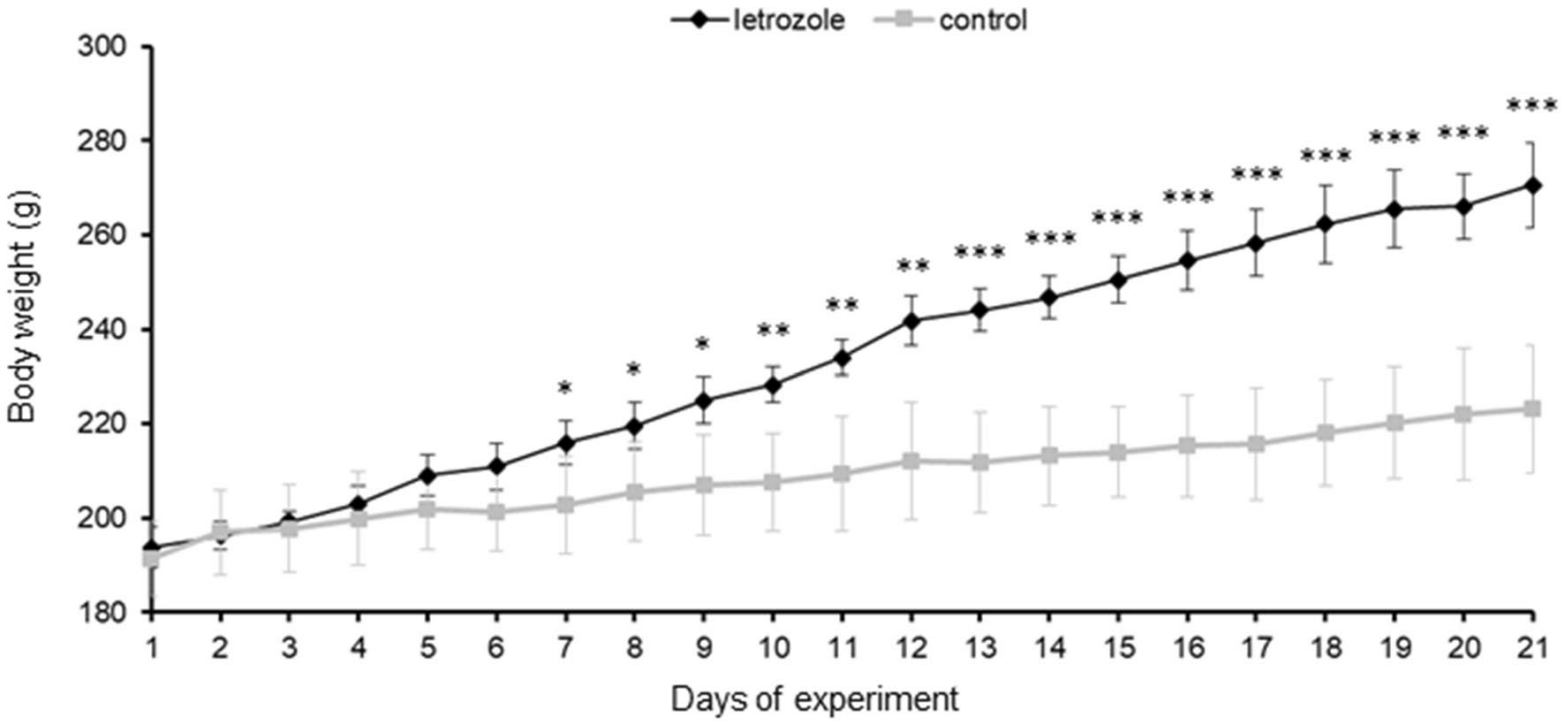
Daily changes in body weight during the course of experiment in control (*n* = 16) and letrozole-treated (*n* = 8) rats. Values are expressed as mean ± SEM *p < 0.05, **p < 0.01, ***p < 0.001 (Student’s *t* test)

Letrozole-treated rats gained more weight than controls (Fig. 1). Statistically significant differences in BW between control and letrozole-treated rats appeared from day 7 (*p* < 0.05) and gradually increased over the course of the experiment (*p* < 0.001).

### Effect of letrozole administration on steroid concentrations

**Fig. 2:**
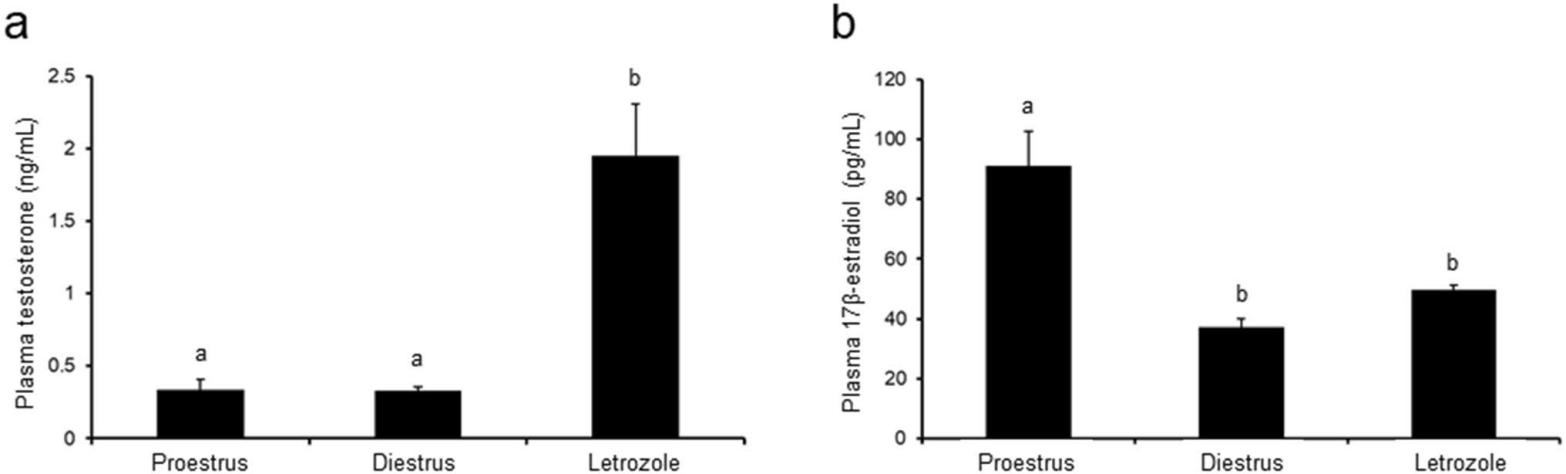
Plasma concentrations of testosterone and 17β-estradiol in control (proestrus and diestrus; *n* = 8 per each group) and letrozole-treated (*n* = 8) rats. Values are expressed as mean ± SEM. Different letters denote statistically significant differences (*p* < 0.05; one-way ANOVA followed by Tukey post hoc test)

Plasma T concentration significantly increased (*p* < 0.05) in letrozole-treated rats when compared to both control groups (Fig. 2a). Plasma E_2_ concentration markedly decreased (*p* < 0.05) following letrozole administration in comparison to the proestrus control group, and was similar to that in diestrus control rats (Fig. 2b).

### Effect of letrozole administration on estrous cyclicity and ovarian histology

**Fig. 3:**
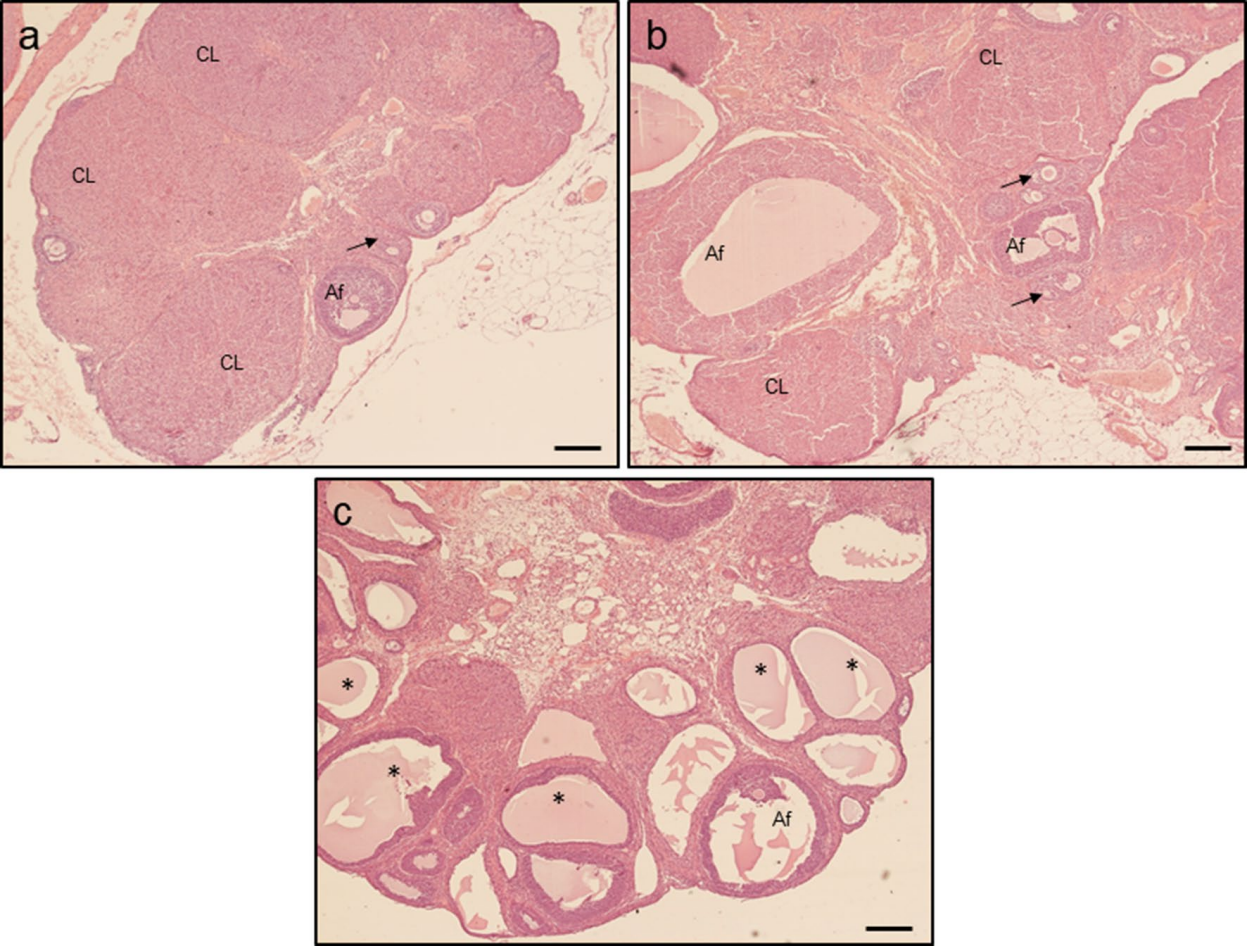
Histology of ovaries obtained from control (**a**—proestrus and **b** – diestrus; *n* = 3 per each group) and letrozole-treated (**c**; *n* = 3) rats. *Af* antral follicle, *CL* corpus luteum, asterisks – ovarian cysts; arrows – preantral follicle. Scale bars represent 200 µm

Animals in both control groups had normal estrous cycle, while rats treated with letrozole were acyclic. Control rats in proestrus (Fig. 3a) and diestrus (Fig. 3b) displayed normal follicular development (preantral follicles, antral follicles and corpora lutea), whereas letrozole administration resulted in ovarian cysts and anovulation confirmed by the lack of corpora lutea (Fig. 3c).

### Effect of letrozole administration on adipocyte size in POAT

**Fig. 4:**
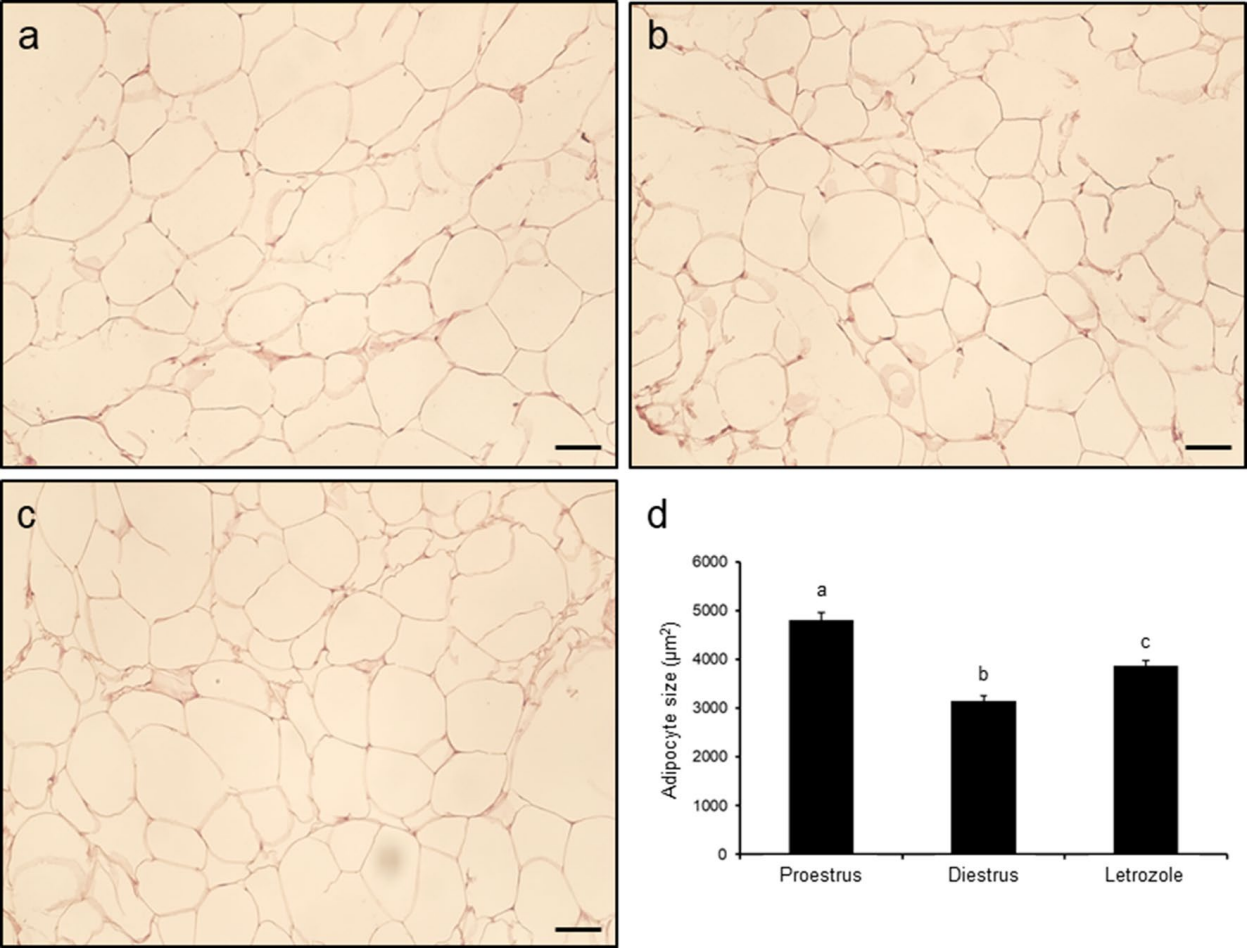
Histology of periovarian adipose tissue obtained from control (**a **proestrus and **b** – diestrus; *n* = 4 per each group) and letrozole-treated (**c**; *n* = 4) rats. Chart (**d**) represents adipocyte size in each examined group. Values are expressed as mean ± SEM. Different letters denote statistically significant differences (*p* < 0.05; one-way ANOVA followed by Tukey post hoc test). Scale bars represent 50 µm

The average size of adipocytes in POAT was greatest in proestrus control rats (*p* < 0.001; Fig. 4a, d), less in the letrozole-treated group (*p* < 0.05; Fig. 4c, d) and least in diestrus control rats (*p* < 0.05; Fig. 4b, d).

### Effect of letrozole administration on plasma 25OHD and calcium concentrations, and 1,25(OH)_2_D_3_ tissue concentration

**Fig. 5:**
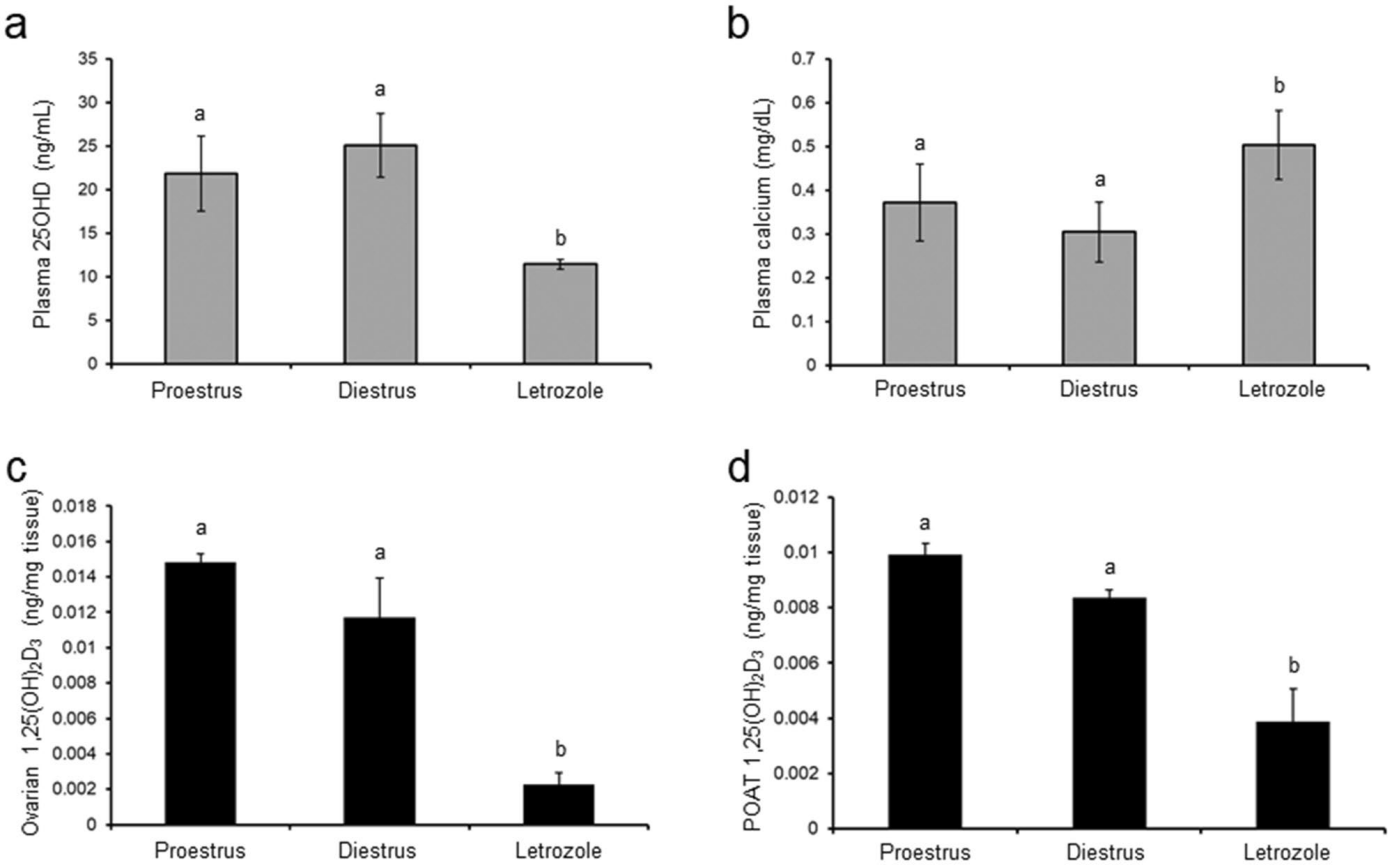
Plasma concentrations of 25OHD (**a**) and calcium (**b**), and 1,25(OH)_2_D_3_ in homogenates of ovaries (**c**) and periovarian adipose tissue (POAT; **d**) in control (proestrus and diestrus; *n* = 8 per each group) and letrozole-treated (*n* = 8) rats. Values are expressed as mean ± SEM. Different letters denote statistically significant differences (*p* < 0.05; one-way ANOVA followed by Tukey post hoc test)

Plasma 25OHD concentration decreased markedly (*p* < 0.05; Fig. 5a) in the group treated with letrozole in comparison to both control groups, whereas calcium concentration did not vary between the groups (*p* < 0.05; Fig. 5b). Neither 25OHD nor calcium level differed between proestrus and diestrus phases (Fig. 5a, b).

The 1,25(OH)_2_D_3_ concentration in homogenates of ovarian tissue (*p* < 0.01; Fig. 5c) and POAT (*p* < 0.05; Fig. 5d) was lower in the letrozole-treated group than in both proestrus and diestrus controls.

### Effect of letrozole administration on *Vdr, Cyp27b1* and *Cyp24a1* mRNA expression in the ovary and POAT

**Fig. 6:**
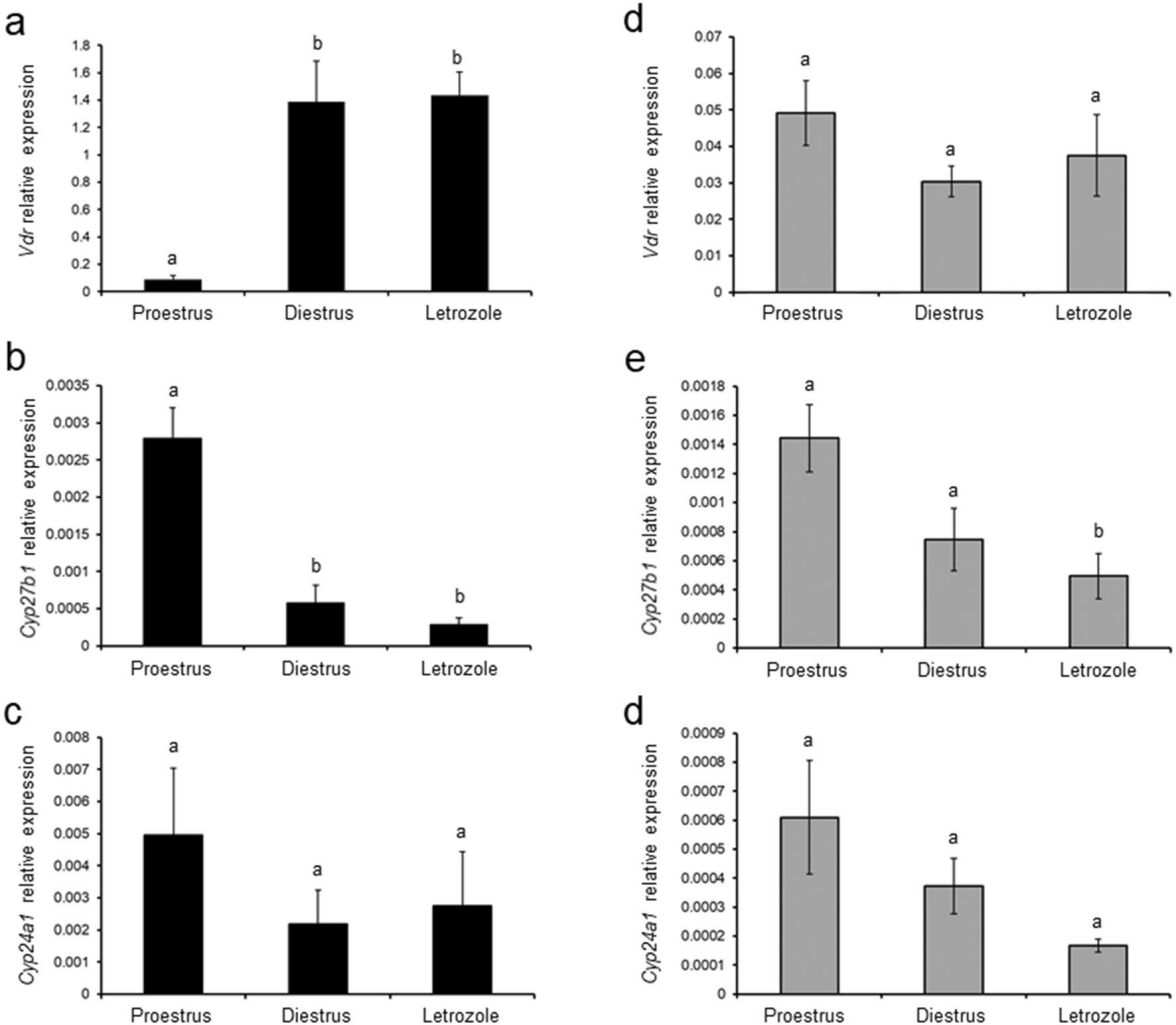
Relative expression of *Vdr* (**a**, **d**), *Cyp27b1* (**b**, **e**) and *Cyp24a1* (**c**, **f**) mRNAs in ovaries (**a–c**) and periovarian adipose tissue (**d–f**) obtained from control (proestrus and diestrus; *n* = 8 per each group) and letrozole-treated (*n* = 8) rats. The mRNA expression was determined using quantitative real-time PCR and presented relative to *Gapdh* as mean ± SEM. Different letters denote statistically significant differences (*p* < 0.05; Kruskal–Wallis test followed by post hoc Dunn's multiple comparison test)

The expression of ovarian *Vdr* mRNA was greater in rats treated with letrozole and control rats in diestrus than in the proestrus control group (*p* < 0.05; Fig. 6a). *Cyp27b1* mRNA expression was lower in the ovaries of the letrozole-treated group and diestrus control group when compared with control rats in proestrus (*p* < 0.05; Fig. 6b), whereas the expression of *Cyp24a1* mRNA was unchanged (Fig. 6c).

In POAT, only *Cyp27b1* mRNA expression markedly decreased following letrozole administration in comparison with both control groups (*p* < 0.05; Fig. 6e), while expression of *Vdr* (Fig. 6d) and *Cyp24a1* (Fig. 6f) mRNAs did not differ between control and letrozole-treated groups.

### Effect of letrozole administration on VDR, CYP27B1 and CYP24A1 protein abundance in the ovary and POAT

**Fig. 7:**
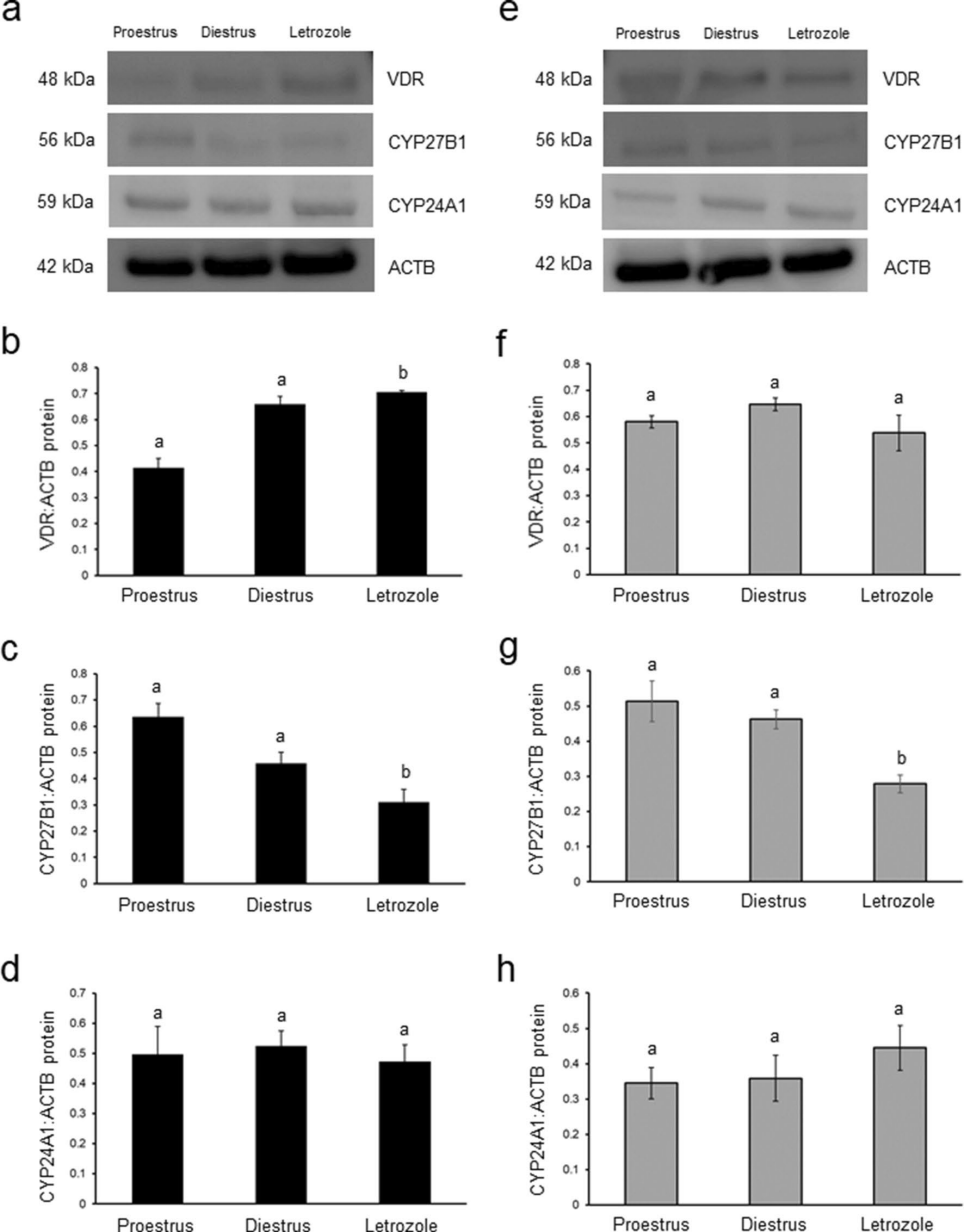
Relative abundance of VDR (**b**, **f**), CYP27B1 (**c**, **g**) and CYP24A1 (**d**, **h**) proteins in ovaries (**b–d**) and periovarian adipose tissue (POAT; **f–h**) obtained from control (proestrus and diestrus; *n* = 8 per each group) and letrozole-treated (*n* = 8) rats. Each protein abundance was evaluated densitometrically and expressed as the ratio relative to β-actin (ACTB) abundance (mean ± SEM). The fragment of membrane with bands corresponding to predicted molecular weights are shown (**a**—ovary; **e**— POAT). Different letters denote statistically significant differences (*p* < 0.05; Kruskal–Wallis test followed by post hoc Dunn's multiple comparison test)

In the ovary (Fig. 7a) and POAT (Fig. 7e) of control and letrozole-treated rats, analyzed proteins were shown as bands at approximately 48 kDa (VDR), 56 kDa (CYP27B1) and 59 kDa (CYP24A1).

The ovarian VDR protein abundance significantly increased (*p* < 0.05; Fig. 7b), while CYP27B1 protein markedly decreased (*p* < 0.05; Fig. 7c) following letrozole administration when compared to both control groups. CYP24A1 protein abundance was at the same level in letrozole-treated rats and controls (Fig. 7d).

In POAT, diminished abundance of CYP27B1 protein was only observed in rats treated with letrozole (*p* < 0.05; Fig. 7g), while VDR (Fig. 7f) and CYP24A1 (Fig. 7h) protein abundances were unchanged between letrozole-treated and control groups.

### Immunolocalization of VDR, CYP27B1 and CYP24A1 in the ovary and POAT

**Fig. 8:**
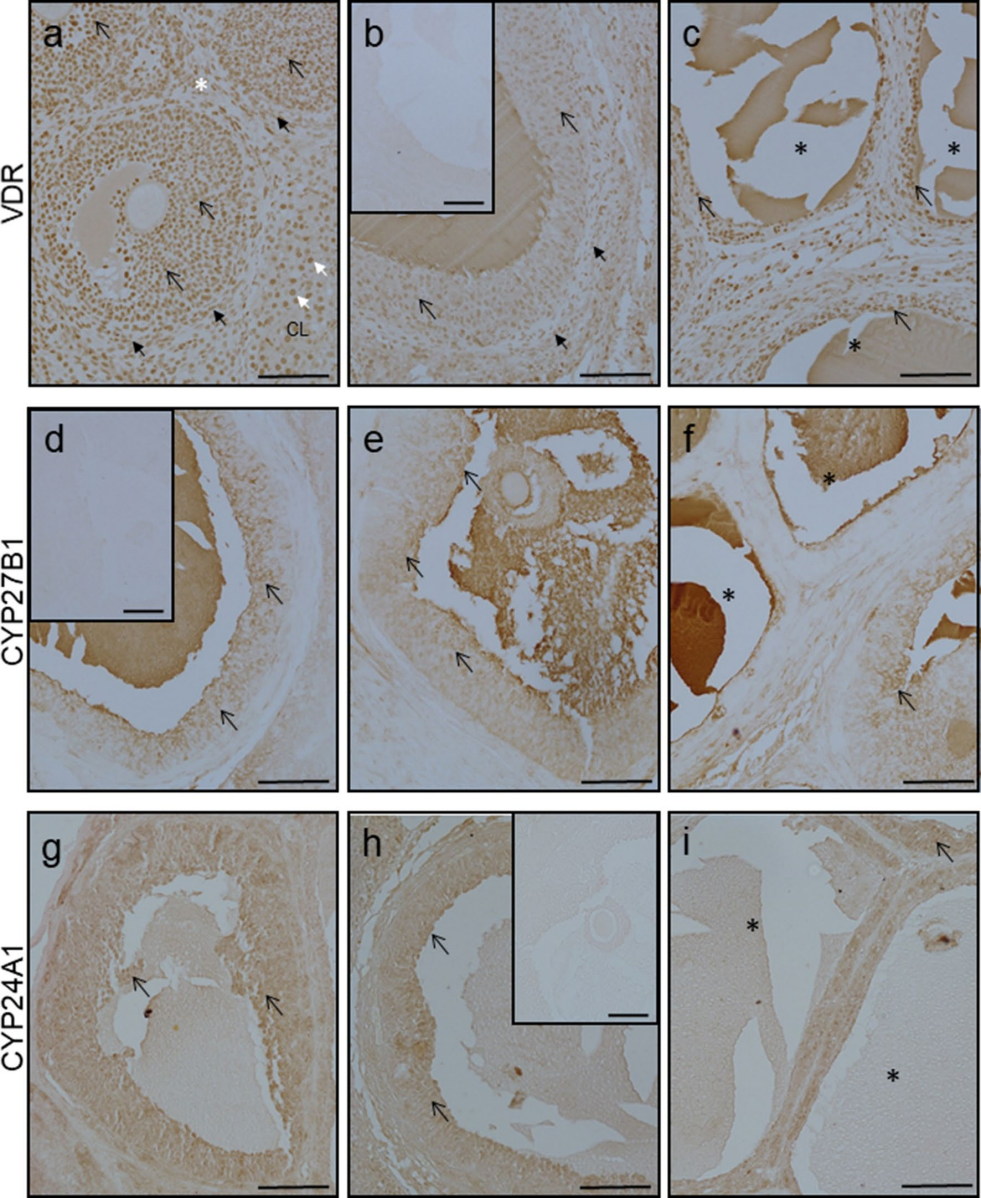
Representative micrographs of immunohistochemical VDR, CYP27B1 and CYP24A1 localization in ovaries obtained from control (**a**, **d**, **g**—proestrus and **b**, **e**, **h**—diestrus; *n* = 3 per each group) and letrozole-treated (**c**, **f**, **i**; *n* = 3) rats. VDR expressed nuclear pattern of staining (**a–c**), while CYP27B1 (**d**–**f**) and CYP24A1 (**g–i**) were observed in the cell cytoplasm. Control sections showed no positive staining (**b** inset, **d** inset, **h** inset). *CL* – corpus luteum; open arrow – granulosa cells; black arrows – theca cells; white arrows – luteal cells; asterisks – ovarian cysts. Scale bars represent 100 µm

**Fig. 9:**
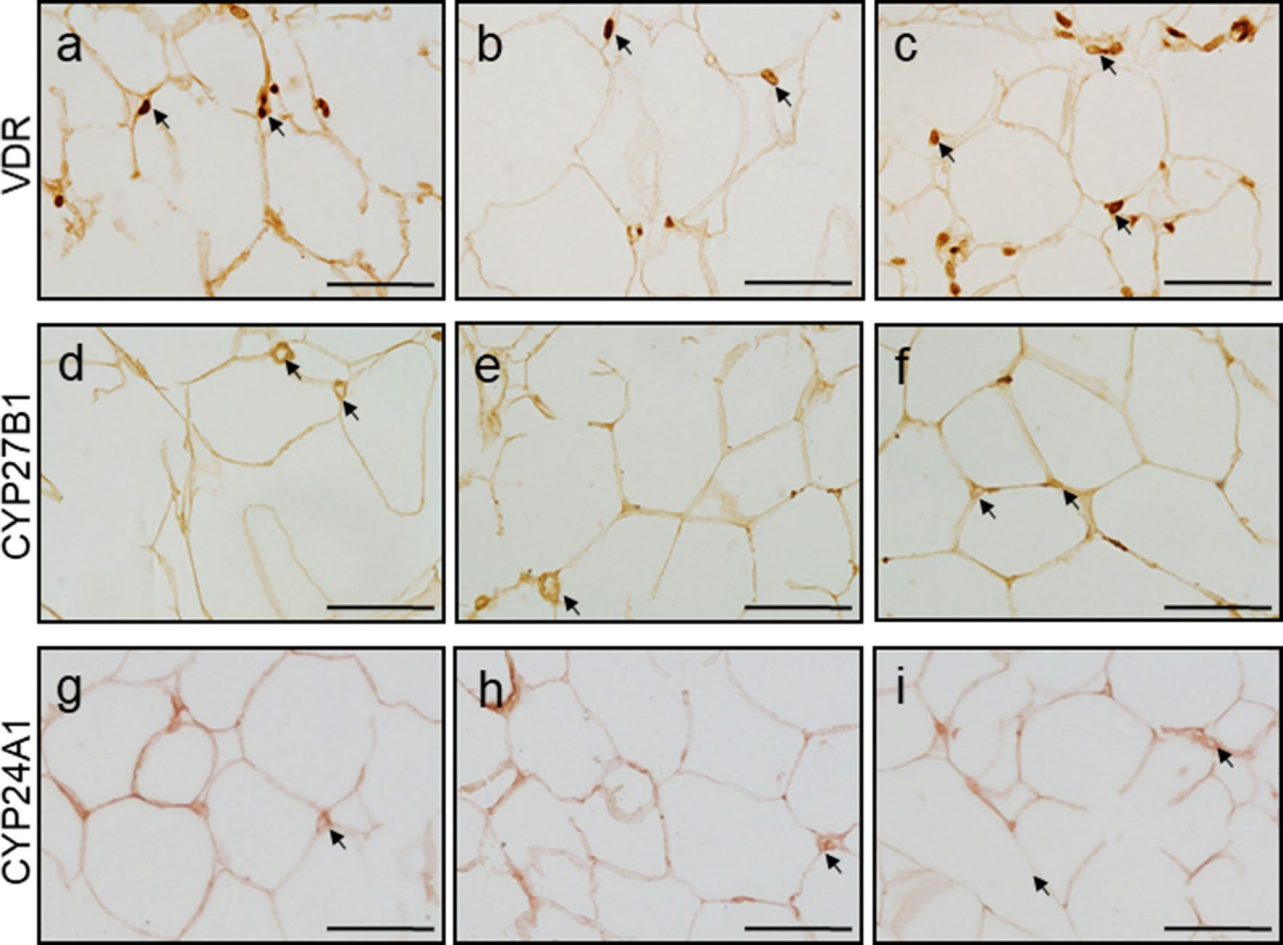
Representative micrographs of immunohistochemical VDR, CYP27B1 and CYP24A1 localization in periovarian adipose tissue obtained from control (**a**, **d**, **g**—proestrus and **b**, **e**, **h**—diestrus; *n* = 3 per each group) and letrozole-treated (**c**, **f**, **i**; *n* = 3) rats. VDR was observed in the nuclei of adipocytes (**a–c**; arrows), while CYP27B1 (**d**–**f**; arrows) and CYP24A1 (**g–i**; arrows) were found in adipocytes cytoplasm. Scale bars represent 50 µm

Positive nuclear VDR staining was found in both ovarian (Fig. 8) and POAT (Fig. 9) sections from the letrozole-treated group (Figs. 8c and 9c) as well as control in proestrus (Fig. 8a and Fig. 9a) and diestrus (Figs. 8b and 9b). In the control ovaries (Fig. 8a, b), VDR immunoreaction was found in granulosa and theca cells of preantral and antral ovarian follicles, and in luteal cells of corpora lutea. In the letrozole-treated group (Fig. 8c), VDR was detected in cells within the luteinized wall of follicular cysts. In POAT, VDR was observed in the nuclei of adipocytes in all studied groups (Fig. 9a–c).

Both metabolizing enzymes, CYP27B1 and CYP24A1, displayed cytoplasmic localization in ovarian (Fig. 8) and POAT (Fig. 9) sections. In the ovary, CYP27B1 (Fig. d-f) and CYP24A1 (Fig. 8g–i) were found exclusively in the granulosa cells of healthy follicles with a lack of positive staining in follicular cysts (Fig. 8f, i). CYP27B1 (Fig. 9d–f) and CYP24A1 (Fig. g–i) were positively localized in the cytoplasm of adipocytes in all examined groups. Either in ovaries (Fig. 8) or POAT (Fig. 9), CYP24A1 immunostaining was weaker than CYP27B1 immunoreaction.

## Discussion

Recently a growing body of evidence highlights that VD_3_ plays an important role in the ovary and its deficiency is linked with several ovarian pathologies such as PCOS. Keeping in mind the expression of CYP27B1 responsible for active VD_3_ synthesis and VDR in ovarian tissue, the question arises whether VD_3_ metabolism at the ovarian level is disrupted in PCOS. Therefore, the present study has been undertaken to examine the local VD_3_ metabolism and the expression of VD_3_-related molecules (VDR, CYP27B1 and CYP24A1) in ovarian tissue and POAT of control rats (at proestrus and diestrus) and those with PCOS induced by the aromatase inhibitor letrozole.

Herein, letrozole-treated rats exhibited key PCOS traits, including acyclicity, anovulation confirmed by the lack of corpora lutea, cystic ovarian morphology and increased BW that is in agreement with previous observations on rodents exposed to letrozole (Kafali et al. 2004; Baravalle et al. 2006; Mannerås et al. 2007; Caldwell et al. 2014). Furthermore, administration of letrozole led to the expected increase in T and decrease in E_2_ concentrations in plasma, reflecting the blockage of androgen conversion to estrogen (Baravalle et al. 2006; Mannerås et al. 2007). Thus, the aforementioned results indicate the successful induction of PCOS rat model in the current study.

It is proposed that VD_3_ deficiency correlates with the occurrence of PCOS. Based on epidemiologic studies, its prevalence in PCOS women is approximately 67–85% (He et al. 2015). In accordance with those results, in PCOS rats we observed decreased concentration of plasma 25OHD. Although lower VD_3_ level is common among PCOS patients, previous research reported a lack of statistically significant differences in VD_3_ status between PCOS and non-PCOS women (Moini et al. 2015). It is noteworthy that a markedly diminished 25OHD level was found in obese compared with lean PCOS patients, suggesting that hypovitaminosis D_3_ results from obesity but is independent of the presence of PCOS (Yildizhan et al. 2009). In this context, the increased BW of letrozole-treated rats observed here might be related to their diminished plasma 25OHD level. It is known that abdominal obesity contributes to the development of insulin resistance in PCOS women, and a positive association between VD_3_ deficiency and this metabolic disorder was also indicated (Yildizhan et al. 2009; He et al. 2015). VD_3_ was shown to enhance insulin synthesis and release, increase insulin receptor expression and inhibit the production of proinflammatory cytokines that might counteract the development of insulin resistance (Sung et al. 2012). In turn, insulin increases ovarian androgen synthesis aggravating hyperandrogenism (Sam and Dunaif 2003). Thus, VD_3_ deficiency might play vital role in the development of subsequent metabolic and endocrine disorders in PCOS.

It is thought that VD_3_ deficiency in PCOS affects calcium homeostasis, resulting in inhibition of oocyte resumption and follicular arrest (Thys-Jacobs et al. 1999). To support this hypothesis, several reports indicated positive effects of VD_3_ and calcium supplementation on menstrual irregularity and infertility in PCOS patients with low VD_3_ status (Thys-Jacobs et al. 1999; Firouzabadi et al. 2012; Kadoura et al. 2019). On the other hand, there were no differences in calcium level between control and PCOS women (Thys-Jacobs et al. 1999; Moini et al. 2015). In agreement with those findings, we have observed no significant changes in plasma calcium concentrations between PCOS rats and control groups. Thys-Jacobs et al. (1999) indicated that calcium metabolism was disrupted in PCOS patients with VD_3_ deficiency and hyperparathyroidism despite normal extracellular calcium concentration. They suggested that dysregulation in intracellular calcium signaling might affect oocyte development and ovulation. However, further studies are needed to confirm this.

Recent studies on humans (Masjedi et al. 2020) and on a PCOS mouse model (Bakhshalizadeh et al. 2017, 2018) have reported improved steroidogenesis and enzymatic antioxidant defense in the granulosa cells isolated from polycystic ovaries following VD_3_ treatment. This suggests a critical role of VD_3_ in establishing proper follicular function in PCOS and that an altered VD_3_ metabolic homeostasis is possible in polycystic ovaries. Disturbed VD_3_ metabolism has been demonstrated in reproductive disorders such as endometriosis (Viganò et al. 2006), preeclampsia (Ma et al. 2012) and ovarian carcinoma (Brożyna et al. 2015), but not in PCOS. The present study shows for the first time a significant reduction of intraovarian 1,25(OH)_2_D_3_ concentration in letrozole-treated rats when compared with controls in proestrus and diestrus. That is probably due to diminished ovarian expression of CYP27B1, which is responsible for the synthesis of active VD_3_, at mRNA and corresponding protein levels in that group. Furthermore, positive CYP27B1 immunostaining was found only in the granulosa cells of healthy follicles, but not in cystic ones. Besides indicating that the rat PCOS ovary is a site of local VD_3_ synthesis, we have now demonstrated its capacity for 1,25(OH)_2_D_3_ catabolism by revealing CYP24A1 expression and immunolocalization. In the ovary of PCOS-induced rats, the expression of mRNA and abundance of protein for CYP24A1 were unchanged. Therefore, our results demonstrate that ovarian conversion of the circulating 25OHD_3_ to active 1,25(OH)_2_D_3_ is decreased in PCOS rat model, and this may contribute to VD_3_ deficiency observed in PCOS.

It is well known that VD_3_ regulates ovarian function through its cognate receptor VDR, expressed abundantly in the mammalian ovary (Herian et al. 2018; Xu et al. 2018). The present immunohistochemical results demonstrate VDR distribution in granulosa and theca cells of healthy rat ovarian follicles, consistent with Johnson et al. (1996). In addition, VDR was detected in the luteinized wall of the follicular cysts. *Vdr* mRNA expression was greater in the ovary of letrozole-treated rats and control rats in diestrus than in proestrus control, whereas VDR protein abundance was increased only in the PCOS group. *VDR* gene is known to be up-regulated by various hormones including parathormone, retinoic acid, glucocorticoids and calcitriol due to their action in the *VDR* promoter region (Pike and Meyer 2010). There are also studies on human ovarian cancer cells (Ahonen et al. 2000) and breast cancer cells (Escaleira et al. 1993) indicating increased VDR protein abundance in response to dihydrotestosterone in vitro. Based on these results, a similar mechanism might exist in PCOS under hyperandrogenic conditions, explaining the increased ovarian VDR protein level following letrozole treatment seen here. Furthermore, androgens were shown to regulate VDR signal transduction through suppression of VDR/retinoid X receptor (RXR) dimerization in porcine ovarian follicles (Herian et al. 2018). Thus, the altered response of ovarian cells to VD_3_ in PCOS, independently of the VDR protein level, cannot be excluded.

Recent research clearly demonstrates that white adipose tissue surrounding the ovaries controls ovarian function through paracrine interactions (Yang et al. 2018). In POAT-deficient mice, the overall estrogens and gonadotropin levels were altered with consequent disturbance to the estrous cycle (Wang et al. 2017). Furthermore, removal of POAT resulted in delayed antral follicle development, increased number of atretic follicles, decreased intraovarian steroidogenic enzyme expression and lipid accumulation, which might reduce the availability of substrate for local steroid production (Yang et al. 2018). Adipose tissue is well known as a large endocrine tissue that can also store and metabolize VD_3_ (Abbas 2017). However, information on VD_3_ metabolism in gonadal adipose tissue is scarce. Recent data showed hypertrophied adipocytes and enhanced inflammation in POAT under dietary restriction of VD_3_ in a mouse model of menopause (Borges et al. 2020), indicating a link between VD_3_ deficiency and POAT function. Considering the role of POAT in ovarian processes, its endocrine function and the occurrence of hypovitaminosis D_3_ in PCOS women, an examination of VD_3_ metabolism in gonadal adipose tissue around polycystic ovaries appears to be required.

The expression of VD_3_-related molecules, including CYP27B1 and CYP24A1, and VDR has been previously described in adipose tissue (Abbas 2017). However, our current research demonstrates for the first time *Cyp27b1*, *Cyp24a1* and *Vdr* mRNA expression as well as CYP27B1, CYP24A1 and VDR protein abundance and immunolocalization in rat POAT. In addition, we have found reduced 1,25(OH)_2_D_3_ concentration in POAT obtained from PCOS rats in comparison with control groups. Accordingly, rat POAT is a local site of VD_3_ metabolism. These results are consistent with previous studies showing the production of active VD_3_ by visceral and subcutaneous adipose tissue (Abbas 2017). The diminished amount of 1,25(OH)_2_D_3_ in POAT of letrozole-treated rats was probably due to the decreased *Cyp27b1* mRNA expression and CYP27B1 protein abundance observed in this group. Notably, we did not find any changes in CYP24A1 and VDR expression at mRNA and protein level between studied groups. In adipose tissue, *CYP27B1* level is lower in obese than lean women, whereas *CYP24A1* expression does not differ between them (Wamberg et al. 2013) explaining obesity associated VD_3_ deficiency. This seems to apply to VD_3_ deficiency in PCOS accompanied by obesity. It is plausible that obese people degrade more VD_3_ in their adipose tissue via CYP24A1 than lean ones. Thus, these data could support the hypothesis of an increased catabolism of VD in obesity besides unchanged CYP24A1 expression (Vranić et al. 2019). Obesity results from the increase in adipocyte number and size (Abbas 2017). Herein we have noted adipocyte hypertrophy in letrozole-treated animals when compared with the control in diestrus, but not when compared with the proestrus control. There are discrepancies between literature reports on the effect of letrozole on adipocyte size in rats. One study revealed enlargement (Mannerås et al. 2007), while another found no change (Caldwell et al. 2014). It should be stressed that neither study was related to POAT.

In conclusion, our results provide novel evidence of disrupted VD_3_ metabolism in the ovary and POAT of rats with induced PCOS. The reduced 1,25(OH)_2_D_3_ concentration in those tissues suggests that they contribute to the VD_3_ deficiency observed in PCOS and might be implicated in PCOS pathogenesis. Furthermore, the letrozole-induced PCOS rat model exhibiting decreased 25OHD plasma level may be valuable for further studies on the role of VD_3_ deficiency in this reproductive disorder.

## Electronic supplementary material

Below is the link to the electronic supplementary material.

Supplementary file1 (DOCX 79 kb)

Supplementary file2 (DOCX 72 kb)

Supplementary file3 (DOCX 206 kb)

Supplementary file4 (DOCX 97 kb)

## Data Availability

Not applicable.
