# Supplementary material for: Altered vitamin D3 metabolism in the ovary and periovarian adipose tissue of rats with letrozole-induced PCOS
Source: Histochem Cell Biol. 2020 Oct 23;155(1):101–16. doi: 10.1007/s00418-020-01928-z (PMC7847874; doi:10.1007/s00418-020-01928-z)

**Supplementary File 1**

Validation of antibodies by Western blot performed on lysates (30 µg of protein) from rat kidney (positive control tissue for VDR, CYP27B1 and CYP24A1) and rat heart (negative control tissue for CYP27B1 and CYP24A1) in comparison to lysates (30 µg of protein) from rat ovary (target tissue). Specific bands of predicted molecular weight were observed as follows: 56 kDa for CYP27B1, 59 kDa for CYP24A1 and 48 kDa for VDR in kidney and ovarian samples. There was no positive reaction for CYP27B1 and CYP24A1 in heart samples. β-actin was used as an endogenous control.


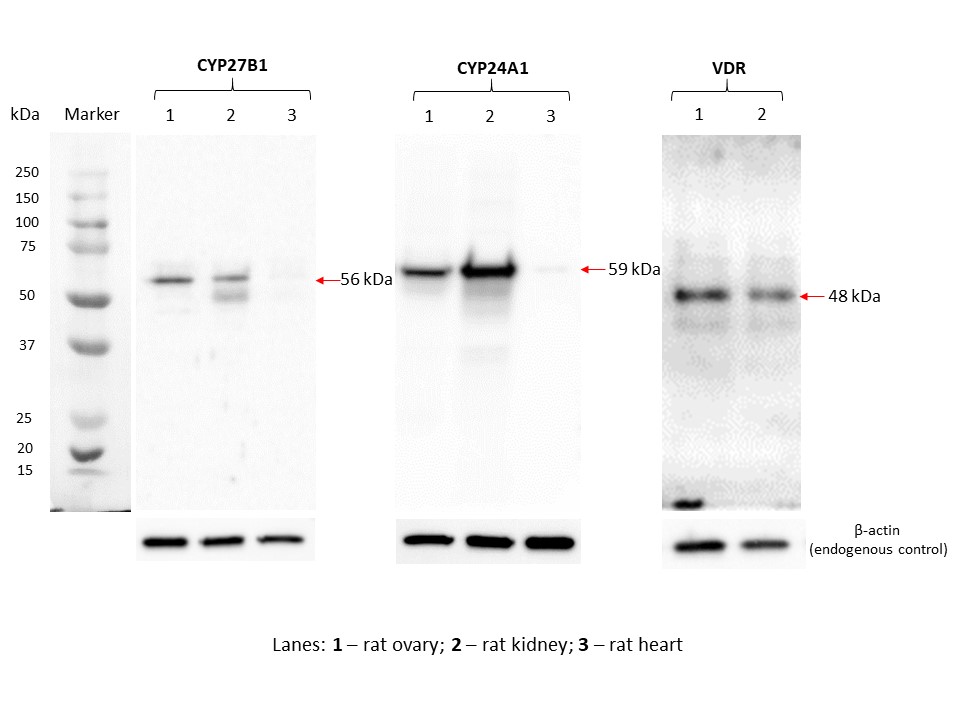

Supplement: Supplementary file 1 — Supplementary file1 (DOCX 79 kb) [file 418_2020_1928_MOESM1_ESM.docx]
