# Supplementary material for: Altered vitamin D3 metabolism in the ovary and periovarian adipose tissue of rats with letrozole-induced PCOS
Source: Histochem Cell Biol. 2020 Oct 23;155(1):101–16. doi: 10.1007/s00418-020-01928-z (PMC7847874; doi:10.1007/s00418-020-01928-z)
Supplement: Supplementary file 2 — Supplementary file2 (DOCX 72 kb) [file 418_2020_1928_MOESM2_ESM.docx]

**Supplementary File 2**

Western blot analysis of non-specific binding of secondary antibodies by omission of primary antibodies. None of analyzed antibodies reveals bands indicating non-specific binding sites.

**
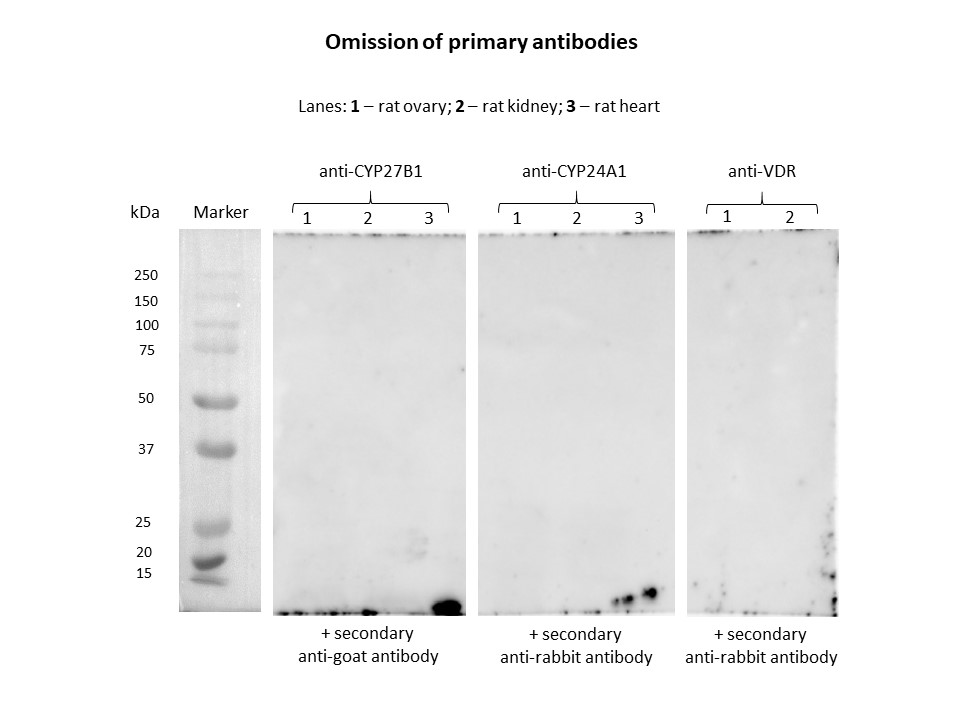
**
