# Supplementary material for: Altered vitamin D3 metabolism in the ovary and periovarian adipose tissue of rats with letrozole-induced PCOS
Source: Histochem Cell Biol. 2020 Oct 23;155(1):101–16. doi: 10.1007/s00418-020-01928-z (PMC7847874; doi:10.1007/s00418-020-01928-z)

**Supplementary File 3**

Validation of primary antibodies by immunohistochemistry on rat kidney sections as a positive control. The incubation with respective non-immune serum (rabbit: NI01 or goat: NI02; Calbiochem, Darmstadt, Germany) was used as a negative control. Positive nuclear reaction was found for VDR, while CYP27B1 And CYP24A1 displayed cytoplasmic pattern of distribution. No positive reaction was observed in negative control sections. Scale bar = 100µm


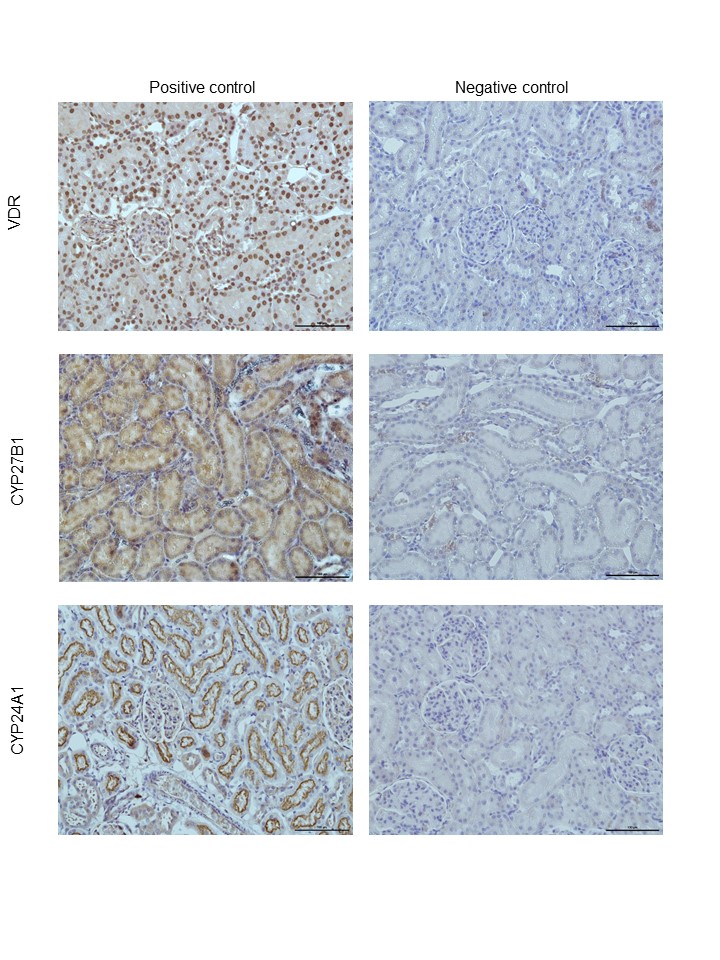

Supplement: Supplementary file 3 — Supplementary file3 (DOCX 206 kb) [file 418_2020_1928_MOESM3_ESM.docx]
