# Supplementary material for: Altered vitamin D3 metabolism in the ovary and periovarian adipose tissue of rats with letrozole-induced PCOS
Source: Histochem Cell Biol. 2020 Oct 23;155(1):101–16. doi: 10.1007/s00418-020-01928-z (PMC7847874; doi:10.1007/s00418-020-01928-z)

**Supplementary File 4**

Antibody validation by employing independent antibody recognizing non-overlapping epitopes of target protein. For VDR protein rat anti-VDR antibody (MA1-710; Thermo Fisher Scientific) recognizing 89 and 105 amino acids sequence of the human VDR and specific for rat was used. It was compared with rabbit anti-VDR antibody (#12550, Cell Signaling Inc.) used in the present study, which recognizes a sequence located surrounding D15 of the human VDR (manufacturer’s proprietary information). For CYP27B1 protein rabbit anti-CYP27B1 antibody (PA5-79128; Thermo Fisher Scientific) recognizing C-terminus sequence between 475 and 508 amino acids of the human CYP27B1 and specific for rat was used. It was compared with goat anti-CYP27B1 antibody (sc-49643, Santa Cruz Biotechnology Inc.) used in the present study, which recognizes a sequence located in the internal region of the human CYP27B1 (manufacturer’s proprietary information). The expression pattern generated by two independent antibodies toward VDR and CYP27B1 proteins yield correlated signals (Pearson correlation was assessed between results presented in the manuscript and results obtained using new antibodies) across loading samples, suggesting that both antibodies recognize the intended target.


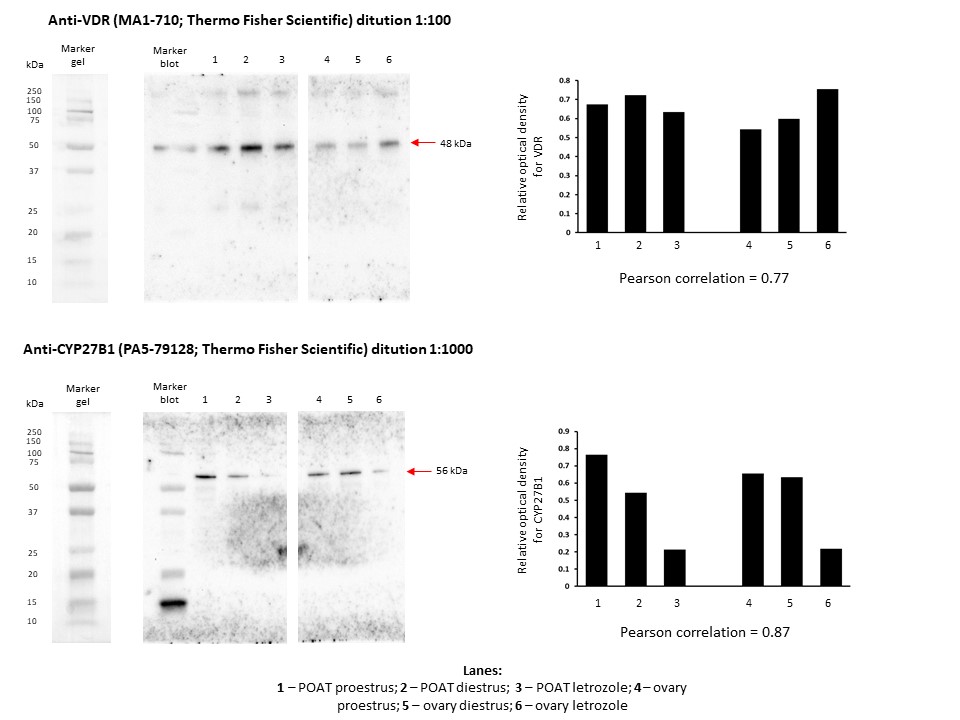

Supplement: Supplementary file 4 — Supplementary file4 (DOCX 97 kb) [file 418_2020_1928_MOESM4_ESM.docx]
